# Supplementary material for: Acute, Sublethal, and Developmental Toxicity of Kratom (Mitragyna speciosa Korth.) Leaf Preparations on Caenorhabditis elegans as an Invertebrate Model for Human Exposure
Source: Int J Environ Res Public Health. 2022 May 22;19(10):6294. doi: 10.3390/ijerph19106294 (PMC9140534; doi:10.3390/ijerph19106294)
Supplement: Supplementary file 1 [file ijerph-19-06294-s001.zip › ijerph-1677387-supplementary.pdf]

International Journal of *Environmental Research and Public Health*

MDPI

Article – Supplemental Material

Acute, Sublethal, and Developmental Toxicity of Kratom (*Mitragyna speciosa* Korth.) Leaf Preparations on *Caenorhabditis elegans* as an Invertebrate Model for Human Exposure

Samantha Hughes [1], David van de Klashorst [2], Charles A. Veltri [3], Oliver Grundmann [3,4,*]

[1] A-LIFE Amsterdam Institute for Life and Environment, Section Environmental Health and Toxicology, Vrije Universiteit Amsterdam, Amsterdam, the Netherlands; s.hughes@vu.nl
[2] BioCentre, HAN University of Applied Sciences, Nijmegen, the Netherlands; d.klashorst@hotmail.com
[3] College of Pharmacy, Department of Pharmaceutical Sciences, Midwestern University, Glendale, AZ 85308, USA; cveltri@midwestern.edu
[4] College of Pharmacy, Department of Medicinal Chemistry, University of Florida, Gainesville, FL 32610, USA; grundman@ufl.edu
* Correspondence: grundman@ufl.edu; Tel.: +1-352-246-4994

Citation: Hughes, S.; van de Klashorst, D.; Veltri, C.A.; Grundmann, O. Acute, Sublethal, and Developmental Toxicity of Kratom (*Mitragyna speciosa* Korth.) Leaf Preparations on *Caenorhabditis elegans* as an Invertebrate Model for Human Exposure. *Int. J. Environ. Res. Public Health* **2022**, *19*, 6294. https://doi.org/10.3390/ijerph19106294

Academic Editor: Piotr Rzymski

Received: 28 March 2022
Accepted: 18 May 2022
Published: 22 May 2022

Publisher's Note: MDPI stays neutral with regard to jurisdictional claims in published maps and institutional affiliations.

Copyright: © 2022 by the authors. Submitted for possible open access publication under the terms and conditions of the Creative Commons Attribution (CC BY) license (https://creativecommons.org/licenses/by/4.0/).

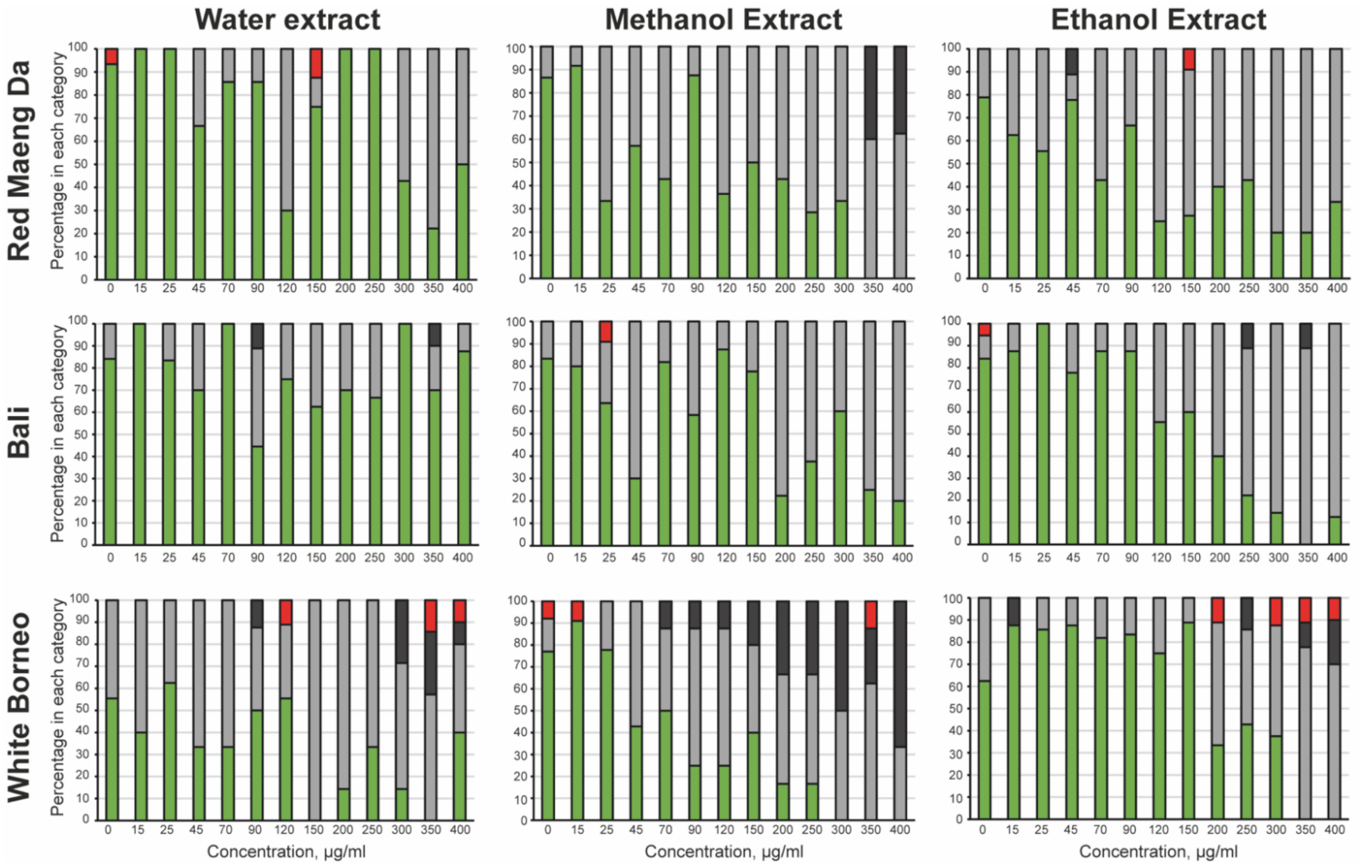

Supplemental Figure S1. Brood size of *C. elegans* after 48 hours incubation with Kratom. Worms were placed in liquid culture at L4 and incubated at 20 °C for 48 hours with gentle shaking at 150rpm. Kratom was added at 15, 25, 45, 70, 90, 120, 150, 200, 250, 300, 350 and 400 µl/mL with White Borneo, Red Maeng Da or Bali varieties extracted in water, methanol or ethanol. The brood size was assessed by binning the data and calculating the percentage of wells in each bin. The stacked bar graphs were then plotted as 'no viable progeny (red bars), 'up to 10 viable progeny' (dark grey bars), '11-75 viable progeny' (light grey bars) or '>75 viable progeny (green bars).

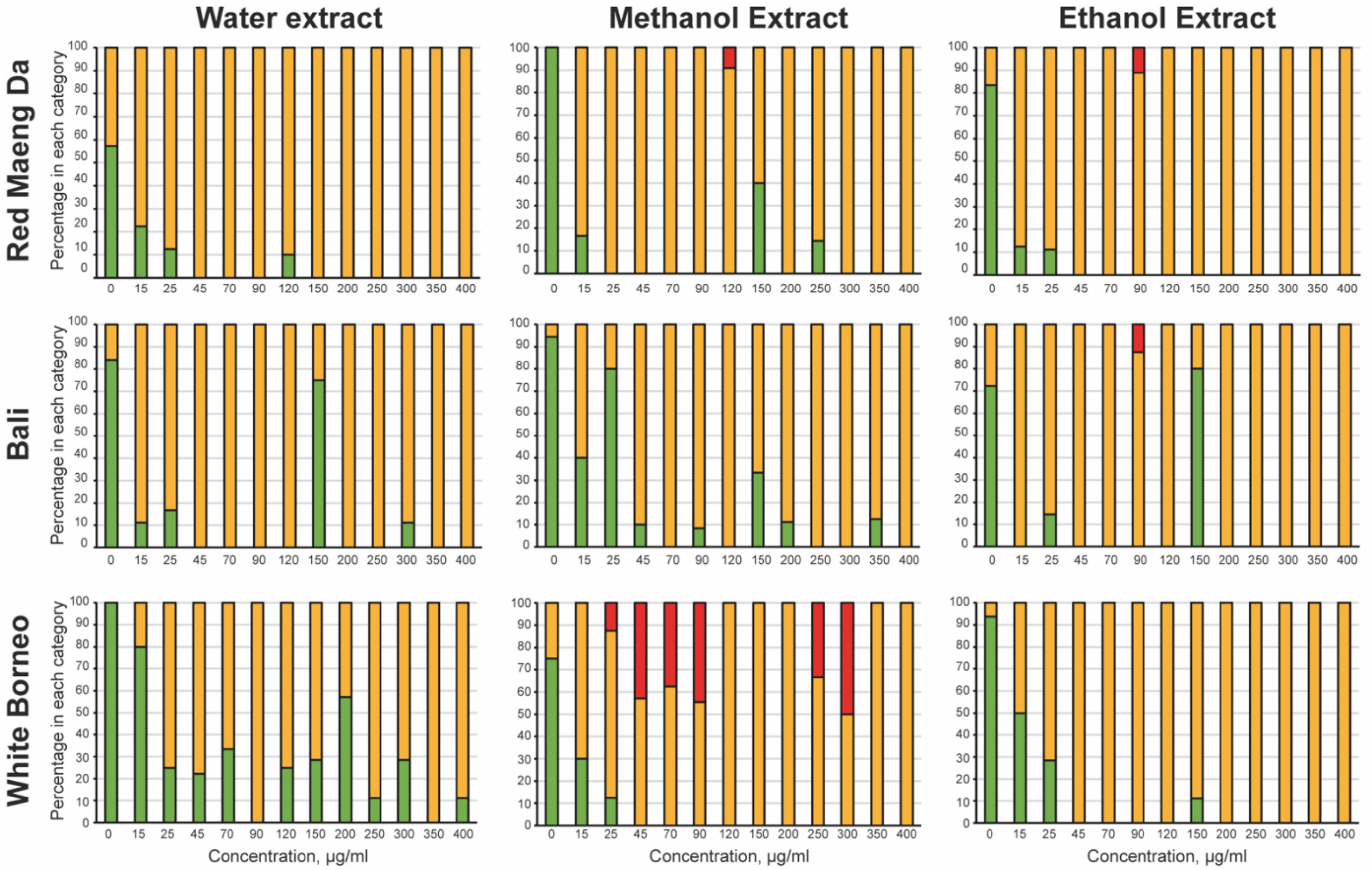

Supplemental Figure S2. Health of the offspring exposed to Kratom. The development of the progeny after 48 hours incubation with the Kratom extracts was classified into 'healthy' (green bars), 'slightly abnormal' (yellow bars) or 'dead/not moving' (red bars). White Borneo, Red Maeng Da or Bali varieties extracted in water, methanol or ethanol at a concentration range of 15, 25, 45, 70, 90, 120, 150, 200, 250, 300, 350 and 400 µg/mL. None of the progeny at any concentration tested was observed to have died. The percentage of wells with offspring in each category, at each concentration is shown.

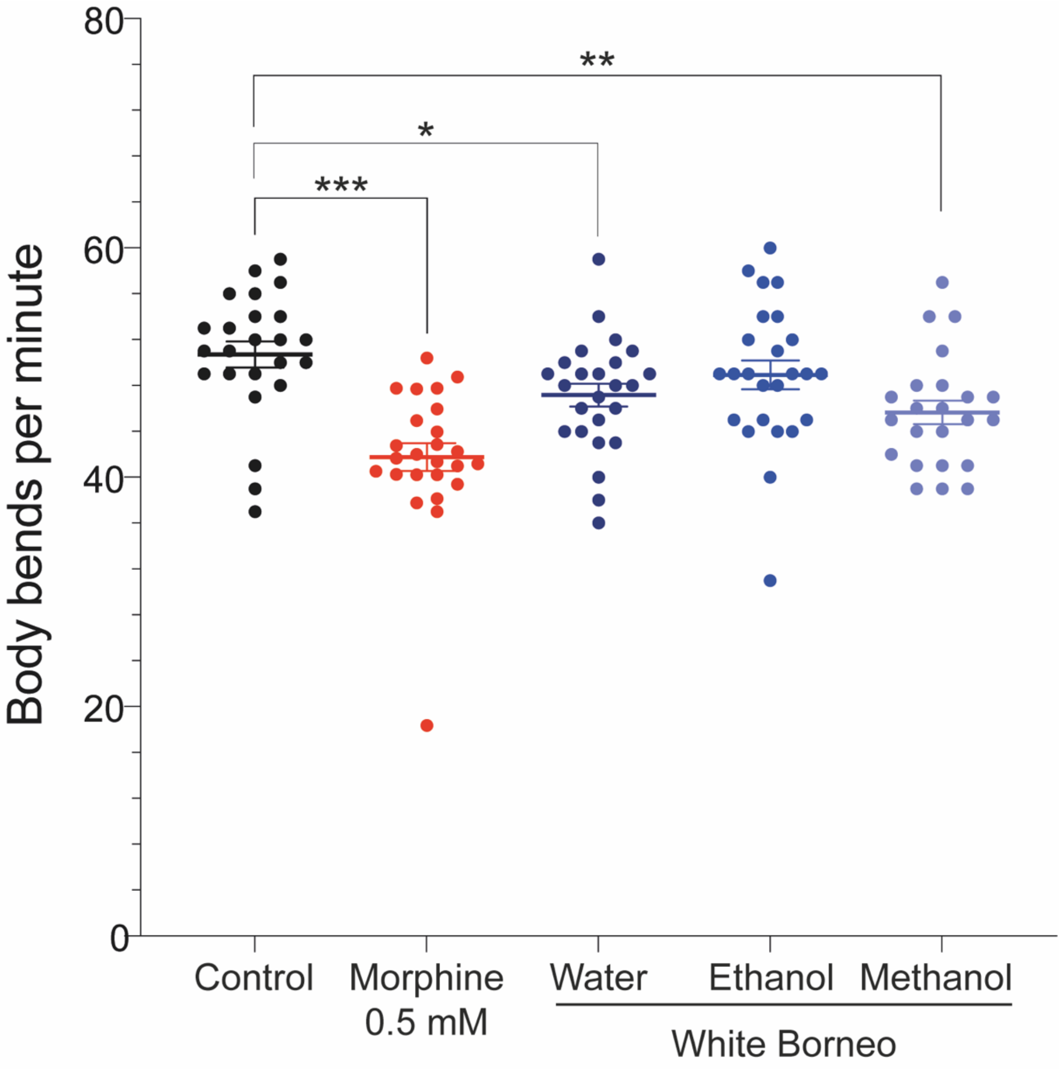

Supplemental Figure S3. Morphine signifcantly reduces thrashing compared to White Borneo kratom extract. Wild type nematodes (strain, N2) were placed on NGM supplemented with Morphine (0.5 mM) or extracts of White Borneo kratom (300 µg/mL) at the L4 stage. After 48 hours, worms were assessed for the number of body bends per minute (BBPM). The data was plotted as average ± standard error of the mean (s.e.m.) with each dot representing a single worm assessed using GraphPad Prism v9. The control worms (black) have an average of 51 BBPM and exposure to morphione signifincatly reduces the BBPM to 40. Water extracts of White Borneo (dark blue) had a slight, but significant reduction of body bends to 47 BBPM while ethanol extracts (blue) had no effect on thrashing with worms have 49 BBPM. Exposure to methanol extracts of White Borneo resulted in an aversge of 46 BBPM, which is signifincatly lower than contros. The statistics were generated using a 2-tailed 2-sample t-test where * shows $p < 0.05$, ** $p < 0.01$ and *** $p < 0.0001$.
